# Gut microbiota-derived imidazole propionate: an emerging target for the prevention and treatment of cardiometabolic diseases

**DOI:** 10.3389/fendo.2025.1409119

**Published:** 2025-02-17

**Authors:** Yan Zeng, Qi Wu, Man Guo, Fangyuan Teng, Chunxia Jiang, Jiao Chen, Xiaozhen Tan, Chen Zeng, Yang Long, Betty Yuen-Kwan Law, Yong Xu

**Affiliations:** ^1^ State Key Laboratory of Quality Research in Chinese Medicine, Dr. Neher’s Biophysics Laboratory for Innovative Drug Discovery, Faculty of Chinese Medicine, Macau University of Science and Technology, Taipa, Macao, China; ^2^ Department of Endocrinology and Metabolism, The Affiliated Hospital of Southwest Medical University, Luzhou, Sichuan, China; ^3^ Metabolic Vascular Diseases Key Laboratory of Sichuan Province, The Affiliated Hospital of Southwest Medical University, Luzhou, Sichuan, China; ^4^ Sichuan Clinical Research Center for Nephropathy, Luzhou, Sichuan, China, The Affiliated Hospital of Southwest Medical University, Luzhou, Sichuan, China; ^5^ Department of Pathology, The Afiliated Hospital of Southwest Medical University, Luzhou, Sichuan, China; ^6^ Experimental Medicine Center, The Affiliated Hospital of Southwest Medical University, Luzhou, Sichuan, China

**Keywords:** imidazole propionate, cardiometabolic disease, diabetes, microbiome, microbial metabolites, biomarker

## Abstract

Despite significant advancements in prevention and treatment, cardiometabolic diseases continue to pose a high burden of incidence and mortality. The chronic progression of these diseases necessitates the identification of early and complementary therapeutic targets to elucidate and mitigate residual risks in patient care. The gut microbiota acts as a sentinel between internal and external environments, transmitting modified risks associated with these factors to the host. Imidazole propionate (ImP), a histidine metabolite originating from the gut microbiota, gained attention after being found to impair glucose tolerance and insulin signaling several years ago. Epidemiological studies over the past five years have demonstrated a robust correlation between ImP and an increased risk of onset of type 2 diabetes (T2D) and obesity, exacerbation of kidney traits in chronic kidney disease (CKD), progression of atherosclerotic plaques, and elevated mortality rates in heart failure (HF). These findings suggest that ImP may serve as a pivotal target for the prevention and treatment of cardiometabolic diseases. Mechanistic insights have uncovered associations between ImP and insulin resistance, impaired glucose metabolism, chronic inflammation, and intestinal barrier damage. This review provides a comprehensive summary of the current evidence regarding the association between ImP and cardiometabolic impairment, highlighting its potential in advancing personalized approaches to disease prevention and management, and exploring the intricate interplay of diet, gut microbiota, and ImP in cardiovascular metabolic impairment. Overall, this review offers valuable insights into the multifaceted roles of ImP in cardiometabolic diseases, identifies current knowledge gaps, and discusses future research directions.

## 1 Introduction

Cardiometabolic diseases impose a significant global health burden, surpassing other disorders in terms of morbidity and mortality, with projections indicating a sharp increase over the next 25 years (1, 2). This category encompasses a range of chronic conditions affecting both cardiovascular and metabolic health, including cardiovascular disease (CVD), insulin resistance, obesity, diabetes, chronic kidney disease (CKD), and nonalcoholic fatty liver disease (NAFLD) (3, 4). Managing these diseases presents challenges for healthcare providers due to their often-asymptomatic nature until advanced stages, highlighting the pressing need for more effective prevention and intervention strategies.

Accumulating evidence implicates imbalances or compositional changes in intestinal microbes in both physiological and pathological alterations in the host (5). The causal contribution of gut microbiota to cardiometabolic diseases is further supported by a plethora of direct experimental evidence (6). A pivotal mechanism involves the production of small molecules by gut microbes, capable of exerting effects at or beyond the host gut barrier. Initially, research primarily focused on bile acids, short-chain fatty acids (SCFAs), branched-chain amino acids, and carnitine-derived metabolites (7–10). Advances in metabolomics have facilitated the identification of increasingly crucial intestinal metabolites, hastening the discovery of potential biomarkers to enhance the diagnosis and prognosis estimation of various diseases.

Recently, imidazole propionate (ImP), a histidine-derived metabolite produced by gut microbes, has garnered increasing attention for its close correlation with metabolic disorder. The investigation into ImP’s role in human disease traces back to 1972 when it was discovered to be excreted by patients with intestinal disorders. Interestingly, it was almost absent in feces and urine from healthy subjects, suggesting ImP’s potential as a microbial metabolite with adverse health effects (11). However, for a considerable period thereafter, ImP seemed to fade into obscurity. It wasn’t until 2018 when researchers from the University of Gothenburg and Sahlgrenska University Hospital (12) discovered its association with impaired insulin signaling in mice and humans that ImP came back into the spotlight. Subsequently, increasing clinical studies reveled close links between circulating ImP levels and metabolic disorder and CVD, including type 2 diabetes (T2D) (13), blood pressure (14), obesity (15), non−alcoholic steatohepatitis (NASH) (16), CKD (17), artery atherosclerosis (18–20), and heart failure (HF) (21). Supplementation with ImP has been demonstrated to exacerbate glucose intolerance (12), impair wound healing (22), and compromise the integrity of the intestinal barrier (23) in mice.

Here, we comprehensively review the available evidence on the biological effects of ImP, emphasizing its potential therapeutic applications as a target for treating cardiometabolic diseases, and discuss future research directions.

## 2 Synthesis and metabolism of ImP in mammals

ImP, also referred to as dihydrourocanate or deamino-histidine, arises from the metabolic activity of gut microbiota on dietary histidine. Histidine, an essential amino acid obtained from the host diet, serves as a fundamental substrate for protein synthesis and acts as a precursor for the biogenic amine histamine, catalyzed by histidine decarboxylase. Moreover, surplus histidine undergoes metabolic conversion to trans-urocanate via histidine ammonia-lyase (EC:4.3.1.3, encoded by the hutH gene) (24). Subsequently, trans-urocanate is primarily metabolized to cis-urocanate in the skin and to glutamate and NH3 in the liver (24, 25). In the colon, urocanate reductase (EC:1.3.99.33, encoded by the urdA gene), produced by the intestinal microbiota, facilitates the reduction of trans-urocanate into the non-metabolizable product, ImP (26, 27). Ultimately, ImP is excreted either directly through feces or absorbed by the intestines and subsequently excreted through urine (11).

Under physiological conditions, circulating ImP levels exhibit minimal individual variation, ranging from a few to several tens of nanomolars (12–14). However, under pathological conditions such as T2D and CVD, its concentration can escalate by approximately a hundredfold (12, 13).

Despite deriving from histidine metabolism, ImP levels in the body are not determined by histidine intake (13), but are primarily affected by enzyme activity and the composition of intestinal microbiota (12, 13, 26). Large-scale screening for UrdA, which encodes the urocanate reductase responsible for ImP production, has identified bacteria harboring “Y” or “M” UrdA homologs as authentic ImP producers from urocanate (12). Urocanate reductase exhibits optimal activity at neutral pH (12, 26, 28). These UrdA-containing bacteria- encompass species such as *Aerococcus urinae*, *Streptococcus mutans*, *Anaerococcus prevotii*, *Adlercreutzia quolifaciens*, *Eggerthella lenta*, *Lactobacillus paraplantarum*, *Brevibacillus laterosporus*, and *Shewanella oneidensis* (12). Over the past five years, an increasing number of intestinal bacteria have been implicated in direct or indirect associations with ImP production, as summarized in **Table 1**.

**Table 1:** Association of microbial features with imidazole propionate production.

Association with ImP	Microbial Features (Indicators or Bacteria)	Reference
ImP-producing bacteria	The bacteria with “Y”- or “M”-UrdA homologs: *Aerococcus urinae, Streptococcus mutans, Anaerococcus prevotii, Adlercreutzia quolifaciens, Eggerthella lenta, Lactobacillus paraplantarum, Brevibacillus laterosporus, Shewanella oneidensis*.	(12)
Positively correlated with ImP levels	Bacteroides 2 enterotype	(13)
	*Lactobacillus*: *L. gasseri, L. fermentum, L. plantarum, L. amylovorus, L. crispatus, L. iners*.	(12, 13, 17, 18, 20, 30, 71, 87)
	*Streptococcus*: *S. mutans, S. parasanguinis, S. gallolyticus, S. anginosus, S. oralis, S. gordonii, S. agalactiae*.	
	*Clostridium*: *C. bolteae, C. symbiosum, C. ramosum, C. scindens, C. bartlettii, C. spiroforme, C. clostridioforme, Flavonifractor plautii*.	
	*Bifidobacterium*: *B. longum, B. dentium, B. breve, B. reuteri*	
	*Bacteroides*: *B. xylanisolvens, B. vatus, B. dorei vulgatus, B. faecis*.	
	Others: *Eggerthella lenta, Veillonella parvula, Veillonella atypica, Dialister invisus, Ruminococcus gnavus, Pseudoflavonifractor capillosus, Gardnerella vaginalis, Peptostreptococcus stomatis, Fusobacterium nucleatum, Parabacteroides gordonii, Blautia hansenii, Citrobacter freundii, Pediococcus acidilactici, Escherichia-Shigella, Allisonella, Collinsella, Desulfovibrio*	
Negatively correlated with ImP levels	Microbiome gene count (607,000 threshold)	(13)
	*Roseburia*: *Roseburia intestinalis, Roseburia hominis*,	(12, 13, 17, 18, 20, 30, 71, 87)
	*Clostridium*: *C. sp. CAG:91, C. sp. CAG:122, C. sp. CAG:127, C. sp. CAG:264*.	
	Others: *Clostridioides difficile, Coprococcus comes, Eubacterium eligens, Faecalibacterium prausnitzii, Dorea formicigenerans, Subdoligranulum variabile, Fournierella massiliensis, Phascolarctobacterium, Anaerostipes, Faecalibacterium, Subdoligranulum variabile*.	

## 3 Role of ImP on cardiometabolic diseases

### 3.1 ImP: linking insulin resistance, type 2 diabetes risk, and metformin inhibition

Bacterial metabolites originating in the gut traverse to the liver via the portal vein before entering systemic circulation. The crosstalk between the gut and liver ultimately leaded to insulin resistance and even diabetes (29). Koh et al. investigated amino acid-derived microbial metabolites potentially linked to insulin resistance and T2D (12). In their initial study involving 15 obese subjects (body mass index [BMI] > 40), higher concentrations of ImP were observed in both portal and peripheral blood of 5 T2D subjects compared to 10 BMI-matched controls. This finding was corroborated in a larger cohort of 649 middle-aged individuals from the Swedish community, where ImP levels remained significantly elevated in treatment-naive T2D subjects after adjusting for BMI, sex, and age (12). Subsequently, numerous studies successively reported the association between ImP levels and T2D. In a large European multicentric cohort (MetaCardis) comprising 1,958 subjects from France, Germany, and Denmark, progressively elevated ImP levels were observed across patients with normal glucose tolerance, prediabetes, and overt T2D (13). Additionally, circulating ImP levels exhibited positive correlations with HbA1C (13, 30), HOMA-IR (13), insulinemia (13), fasting glucose (13, 15) and postprandial glucose (13, 31). These findings indicate that ImP is not only associated with impaired glucose metabolism but also with diabetic status.

The analysis of gut microbiota in T2D patients effectively addressed the reasons for the changes in circulating ImP levels. In an *in vitro* gut simulator experiment monitoring ImP production kinetics, the gut microbiota of T2D patients demonstrated ImP production capability, unlike non-T2D patients (12). Further investigation revealed the enrichment of ImP-producing bacteria, characterized by “Y” or “M” UrdA homologs, in the intestines of T2D patients (12), as well as in the intestine and skin of T2D mice (22). These included strains previously associated with an elevated risk of T2D in large population cohorts, such as *Streptococcus mutans* (32), *Eggerthella lenta* (33), and *Lactobacillus gasseri* (33).

However, ImP serves not only as a disease marker but also correlates with an increased risk of prediabetes and T2D, as demonstrated in cohort studies (13, 15), suggesting its biological impact on T2D progression. In animal experiments, ImP injection induced glucose intolerance and decreased hepatic insulin signaling (12). Mechanistically, ImP disrupts insulin signaling by activating p38γ mitogen-activated protein kinase (MAPK), leading to p62 phosphorylation and subsequent activation of mechanistic target of rapamycin complex 1 (mTORC1). This results in the phosphorylation and degradation of insulin receptor substrates 1 and 2 (IRS1 and IRS2). Consistently, phosphorylation of p62 and S6K1 were elevated in the human liver compared to healthy controls (12), highlighting the role of ImP in impairing insulin signaling through the p62/mTORC1 pathway.

In addition to its impact on T2D itself, ImP has also been found to influence the hypoglycemic effects of metformin, the first-line therapy for T2D. Metformin exhibits substantial variability in efficacy among individuals, and genetic variations, particularly in genes encoding transporters such as organic cation transporter 1 (OCT1) (34) and glucose transporter 2 (GLUT2) (35), have been identified as influencing metformin response. In addition to gene polymorphisms, Koh et al. discovered that intestinal ImP levels contribute to this variability (36). T2D patients on metformin with persistently high blood glucose levels showed elevated ImP concentrations.

Further experimental studies demonstrated that ImP diminishes the glucose-lowering effect of metformin and inhibits metformin-induced activation of adenosine 5′-monophosphate-activated protein kinase (AMPK) by impeding AMPK serine phosphorylation through the p38γ/Akt pathway. However, as the authors point out, the study has certain limitations worth noting. Given its cross-sectional design, it remains unclear whether individuals with higher blood glucose values (and consequently higher plasma ImP levels) actually responded poorly to metformin or had more severe diabetes prior to treatment initiation. Hence, a longitudinal cohort study is warranted to ascertain whether ImP directly undermines metformin efficacy and whether metformin contributes to the proliferation of ImP-producing bacteria.

### 3.2 ImP: implicated in atherosclerosis through chronic systemic inflammation and immune activation

The sub-analysis of the MetaCardis study, including 20% of participants with CVD, revealed a significant increase in circulating ImP concentrations among CVD patients after adjusting for traditional risk factors (age, gender, BMI, ethnicity), kidney function, and presence of T2D (13). Correlation analysis demonstrated a strong positive correlation between ImP levels and serum inflammatory markers, including total leukocyte count, high-sensitivity C-reactive protein (hs-CRP), and interferon gamma-induced protein 10 (IP-10) (13), suggesting a potential association between ImP, inflammation, and CVD progression, warranting further investigation.

Recent large-scale clinical studies have linked ImP to atherosclerosis (18–20), a chronic inflammatory vascular disease and the major cause of CVD (37). The role of gut microbiota in atherosclerosis has been supported by increasing mechanistic evidence (38). In fact, significant changes in gut microbiota have been observed in patients with subclinical coronary atherosclerosis before plaque formation. A study involving 8, 973 participants without overt atherosclerotic disease revealed significant alterations in gut microbiota, including oral microbial species like *Streptococcus* spp, which correlated significantly with ImP levels and systemic inflammation markers (hs-CRP levels and neutrophil counts) (20). However, due to its cross-sectional design and lack of experimental evidence, the study failed to extrapolate the predictive value of ImP on plaque formation and its potential implications in atherogenesis.

Nevertheless, data from HIV patients have provided indications of a possible association between ImP and the presence of plaques in carotid and coronary arteries (18, 19). HIV infection is linked to chronic inflammation and immune activation, critical factors in atherosclerosis and thrombosis development (39). HIV-induced disruptions in gut microbiota exacerbate chronic inflammation and metabolic irregularities, increasing atherosclerosis risk (40, 41).

In a study of 320 females living with or at risk of HIV infection, with 26% having carotid artery plaque, a distinct shift in gut microbiota composition and increased ImP plasma levels were observed (18). Circulating ImP levels were inversely correlated with potentially beneficial microbial species linked to reduced carotid artery plaque. Additionally, ImP levels positively correlated with serum inflammatory markers, including CX3CL1, TNFSRF9, and LIF-R, associated with immune activation and inflammation pathways related to atherosclerotic plaques (42–44). Notably, after adjusting for plasma ImP levels or inflammatory markers, the association between gut bacterial species and plaque weakened, indicating that circulating ImP levels and related inflammatory markers may partly explain these associations. Further analysis identified 17 ImP-associated species (as illustrated in **Table 1**), with 8 correlating with the functional enzyme hutH. A gut microbiota score derived from these ImP-associated species showed a positive correlation with plaque formation and several pro-inflammatory markers, even after adjusting for multiple factors. Collectively, gut bacteria may contribute to plaque formation by modulating host immune activation and inflammation through elevated IMP levels.

Recent research suggests that ImP is not only associated with plaque formation but also with plaque obstruction. HIV-infected patients with obstructive coronary artery disease (CAD) exhibited lower gut microbiota diversity and significant compositional changes compared to HIV-infected individuals without CAD or non-obstructive CAD, with increased abundance of known ImP producers such as *Rumiococcus gnavus* and *Veillonella* (19). ImP plasma levels were associated with this dysbiosis, significantly elevated in participants with obstructive CAD (19). However, after adjustment for traditional and HIV-related risk factors, gut dysbiosis but not plasma ImP was independently associated with obstructive CAD, indicating that the effects of gut microbiota may extend beyond ImP in plaque obstruction. Longitudinal studies are needed to establish a causal relationship between ImP levels and plaque formation, shedding light on its predictive value for atherosclerosis. Additionally, exploring ImP in the context of HIV-related CVD is crucial, highlighting the importance of understanding the role of ImP and gut dysbiosis in driving CVD risk among HIV patients.

The recent findings offer a strong foundation for future investigations, yet additional experiments are necessary to move beyond associations and establish causal evidence linking ImP to atherosclerosis. Firstly, further research is needed to elucidate the mechanistic connection between ImP and inflammation, addressing the significant correlations observed in multiple studies. Secondly, Atherosclerosis initiates with endothelial injury, leading to the accumulation of macrophage foam cells and infiltration of smooth muscle cells, resulting in fatty streak formation (45). Inflammatory processes play a pivotal role in the development of vulnerable plaques (46). Rupture of the plaque’s cap triggers platelet aggregation, precipitating thrombosis and vascular obstruction (47). Therefore, understanding whether ImP is involved in endothelial injury, foam cell formation, and platelet aggregation should be the central focus of future research into the progression of atherosclerosis.

### 3.3 ImP independently predicts incident heart failure and mortality

Individuals with T2D face more than a twofold increased risk of developing HF compared to non-T2D patients, with higher risks of incident cases and mortality among diagnosed patients (48). Profiling the metabolic connections and shared components of T2D and HF could unveil new disease pathways, enhance risk prediction, and enable tailored prevention and management strategies (49). The elevation of intestinal metabolite ImP in the circulation of T2D patients, along with its induction of insulin resistance in animal models (12), offers a novel perspective on unraveling the molecular signatures and metabolic remodeling of HF and its associated factors.

Targeted metabolomic analysis of 260 individuals with diverse glucose metabolism from the Risk Evaluation and Management of Heart Failure (REM-HF) cohort in China identified ImP as a microbial signature contributing to the shared etiologies of T2D, HF, and CKD (21). Data from the Boston Puerto Rican Health Study (BPRHS) cohort (50) and European Prospective Investigation into Cancer (EPIC)-Norfolk study (49) further corroborated this finding. Impressively, serum ImP levels increased by 1.1–1.6 fold with each additional chronic HF comorbidity (21), supporting ImP as a component of the metabolic connections among T2D, HF, and CKD.

Recently, Molinaro et al. from Sweden (51) investigated the association between circulating ImP levels, HF, and incident mortality risk. In the population-based MetaCardis cohort, significantly higher ImP levels were observed in individuals with established CVD or HF compared to those without, with the highest levels detected in HF patients. Individuals in the highest quartile of ImP levels had a threefold increased risk of HF compared to those in the lowest quartile, even after adjusting for multiple traditional cardiovascular risk factors. Moreover, ImP levels were inversely associated with left ventricular ejection fraction (LVEF) and positively correlated with pro-atrial natriuretic peptide (proANP) and N-terminal pro–B-type natriuretic peptide (NT-proBNP) levels. These findings were consistent across the GeneBank cohort from North America, predominantly comprising patients with HF (*n* = 407), CVD (*n* = 1,331), and without CVD or HF (*n* = 417). Additionally, longitudinal follow-up data from the North American cohort revealed that the highest quartile of ImP was independently associated with an increased risk of overall mortality, even after adjusting for traditional risk factors and baseline covariates (adjusted HR = 1.85, 95% CI [1.20, 2.88], *P* < 0.01). Overall, this study, drawing from two large independent cohorts, offers compelling evidence supporting a substantial correlation between ImP levels and CVD, HF, and HF-associated phenotypes including reduced left ventricular ejection fraction and heightened natriuretic peptide levels. Crucially, this correlation persists regardless of obesity and T2D, known contributors to disease progression. *In vitro* experiments conducted on H9c2 cardiomyoblast cells pretreated with hypoxia/reoxygenation further supported a causal link between ImP and distinct HF-relevant phenotypes. In this study, the intervention of 0.1 μM ImP for 24 hours significantly elevated the expression of the Natriuretic Peptide B gene (NPPB), which encodes the B-type natriuretic peptide (BNP), and disrupted cardiomyoblast functions, as indicated by significantly reduced mitochondrial membrane potential (21).

The current research on the association between elevated circulating ImP levels and HF presents valuable insights, yet it also reveals several limitations and areas for future investigation. One major concern is the lack of clarity regarding the underlying reasons for the elevation of ImP in HF patients, posing a significant gap in our understanding. Moreover, the reliance on cross-sectional data underscores the need for longitudinal research to establish definitive causal relationships. Furthermore, the predominantly focused research on specific populations calls for more diverse cohorts to ensure the generalizability of findings. Future studies should prioritize exploring the mechanistic understanding of ImP’s role in HF development and conducting interventional trials to assess the therapeutic potential of targeting ImP levels. Consideration of confounding factors such as medication use and lifestyle variables is essential, and efforts to validate ImP as a diagnostic and prognostic biomarker are warranted. Addressing these gaps through comprehensive research endeavors will enhance our understanding of the involvement of ImP in HF and facilitate the development of effective therapeutic strategies.

### 3.4 ImP: a novel factor associated with nonalcoholic fatty liver disease

NAFLD, affecting approximately one quarter of the global population, encompasses a spectrum of conditions ranging from simple hepatic steatosis, often linked to obesity, to NASH, which can progress to fibrosis, cirrhosis, and hepatocellular carcinoma (52). The gut and liver are interconnected through the portal vein, forming the gut-liver axis, which serves as a direct pathway for gut microbiota and their metabolic by-products to reach the liver (53). Bidirectional communication along the gut-liver axis plays a pivotal role in NAFLD pathogenesis (54). In NAFLD, microbial dysbiosis in the gut, particularly a decrease in SCFAs-producing microbiota, has been documented (55). This reduction in SCFAs production leads to an elevation in intestinal pH (56), influencing bacterial metabolite production and subsequent absorption into the host circulation (57).

In Göttingen minipigs fed a choline-deficient amino acid-defined high-fat diet (CDAHFD), serving as a NASH animal model, notable increases in serum ImP concentration and pancreatic glucagon levels were observed. These changes were accompanied by liver activation of mTORC1, as indicated by increased expression of liver RHEB and MTOR genes, along with impaired hepatic insulin signaling, demonstrated by decreased expression of IRS1 and IRS2 (16). Moreover, multiple linear regression analysis identified ImP as a statistically significant predictor for glucagon levels (*P* = 0.0068). Additionally, 16S rRNA analysis showed significant downregulation of intestinal SCFAs-producing bacteria in CDAHFD-fed minipigs, including butyrate-producing members of *Lachnospiraceae* and propionate producers from the *Muribaculaceae* family (16). This resulted in an elevation of colon luminal pH, creating an environment conducive to enzymatic activity of bacterial urocanate reductase, thereby facilitating ImP production from histidine metabolism (12, 16, 26, 28). Following production, ImP is likely transported from the intestines to the liver via the portal vein (58), where it subsequently contributes to impaired hepatic insulin signaling, hyperglucagonemia, decreased expression of the glucagon receptor, and disruption of the liver-α-cell axis (16). This aligns with emerging evidence showing a negative correlation between ImP levels and fibroblast growth factor 21 (FGF-21), an endogenous regulator of lipid and glucose metabolism (30). Recently, ImP were enriched in cirrhotic patients with chronic hepatitis B compared to healthy subjects, signifying its potential role in chronic hepatitis B progression, but its role remains to be further clarified.

Future research should delve into elucidating the exact role of ImP in the pathogenesis of NAFLD and other liver disease. Specifically, efforts should focus on determining the association of ImP with the progression of liver diseases and its potential diagnostic and therapeutic implications. Moreover, researchers could explore strategies to modulate ImP levels by manipulating gut microbiota composition or intervening in gut SCFAs production, thereby developing novel treatment approaches.

### 3.5 ImP: a promising target for chronic kidney disease

Globally, over 10% of the population suffers from CKD, characterized by kidney damage, typically indicated by urinary albumin, or decreased kidney function, measured by glomerular filtration rate (59). Individuals with CKD face a significantly elevated risk of CVD and cardiovascular-related mortality (60), underscoring the importance of early detection for effective management. The gut microbiota and their associated metabolites play a crucial role in the microbiota-gut-kidney axis, offering a promising avenue for early diagnosis and personalized treatment to slow renal progression (61).

Recent studies have identified ImP as another metabolite linked to kidney traits, prospectively associated with CKD incidence over time (17). In a large study involving 2,438 Hispanic/Latino adults (12% with CKD), elevated ImP levels were correlated with worsening kidney traits, including reduced eGFR, increased urinary albumin-to-creatinine (UAC) ratio, and CKD incidence over approximately 6 years (17). Similarly, in another cohort from China with varying glucose tolerances, ImP levels exhibited a strong association with creatinine, Cystatin C, and estimated glomerular filtration rate (eGFR). Furthermore, serum ImP levels increased by 1.5 times with the occurrence of CKD in patients with T2D and chronic HF (21). Notably, ImP was more strongly associated with biomarkers of CKD than with those of HF and T2D, indicating its potential pathogenic role in all three conditions and suggesting shared etiologies mediated by ImP among these diseases.

The understanding of the mechanisms underlying increased ImP levels in CKD is still evolving. Alterations in the gut microbiota may be a contributing factor, as CKD progression leads to factors such as sodium and water retention, increased circulatory system pressure, visceral congestion, intestinal wall edema, and impaired intestinal barrier function, resulting in bacterial translocation and gut dysbiosis (62). Studies like the Hispanic Community Health Study/Study of Latinos (HCHS/SOL) study (17) have shown that higher CKD incidence rates and UAC ratios, along with lower eGFR, are associated with reduced gut microbiota diversity and alterations in overall microbial composition, which may directly contribute to increased ImP production. Future research should further explore the relationship between CKD progression, gut dysbiosis, and ImP production to uncover potential therapeutic strategies for managing CKD-related complications and reducing the risk of ImP-associated health issues.

Another interesting discovery is the observed prospective association of ImP with changes in renal function and impairment in CKD, particularly prominent in patients with diabetes (17). This is consistent with animal studies where serum ImP levels were significantly elevated in diabetic mice models (63). ImP levels positively correlated with renal functional parameters like the UAC ratio. In cellular studies, ImP was found to stimulate inflammation and fibrosis by promoting toll-like receptor 4 (TLR4)-mediated phosphorylation of NF-κB and Stat3 and expression of IL-6, TGF-β1, and MyD88 (63). Moreover, downregulating ImP-producing bacteria, such as certain genera of *Bacteroides* and *Unidentified Ruminococcaceae*, was shown to ameliorate diabetic kidney disease by suppressing ImP-induced protein expression of the TLR4 signaling pathway *in vivo* and *in vitro* (63). Understanding the mechanistic role of ImP in CKD progression and its associations with other renal conditions, along with validating these mechanisms through extensive cell and animal model studies, could lead to improved clinical applications in the future.

### 3.6 ImP: a potential biomarker for obesity and its association with blood pressure

The interconnection among obesity, hypertension, diabetes, and CVD underscores the importance of investigating shared metabolic pathways to improve risk assessment and develop personalized prevention and management strategies. Studies exploring the association between ImP, T2D, and CVD, such as those by Koh and Molinaro, et al. (12, 13) have included patients with overt metabolic diseases or CVD, complicating the differentiation of ImP’s correlation with known risk factors like weight gain, hypertension, and cholesterol. Insights from microbiome community typing analyses within the MetaCardis cohort provided some clues, revealing elevated ImP levels in individuals with the Bacteroides2 (Bact2) enterotype (13), a gut microbiota profile associated with systemic inflammation and obesity (64). Research involving 1,018 females from the UK Adult Twin Registry (TwinsUK) cohort further supports the correlation between ImP and obesity (15). This study found that serum ImP levels were positively correlated with BMI (Pearson correlation coefficient [*r*] = 0.18, *P* = 5E-9), visceral fat mass (*r* = 0.067, *P* = 0.06), and an increased risk of obesity (*r* = 0.2, *P* = 8E-9), indicating circulating ImP levels as a potential marker for obesity.

In another cohort of overweight/obese subjects without T2D and not on any CVD medication, the association between circulating plasma ImP concentrations and CVD risk factors, including blood pressure, HDL cholesterol, and LDL cholesterol, was investigated (14). This study revealed a positive correlation between plasma ImP concentrations and diastolic blood pressure (Spearman rank correlation coefficient [*rs*] = 0.285, *P* = 0.004), with borderline significance for systolic blood pressure (*rs* = 0.187, *P* = 0.060). Interestingly, no significant association was found between plasma ImP concentrations and peripheral or hepatic insulin resistance, contrary to the findings of Koh et al. (12). This discrepancy could be attributed to differences in the study populations, as Koh et al.’s study included patients with varying BMI and metabolic disease severity. However, the data revealing the association between ImP and blood pressure comes from cohort comprising subjects without T2D, with a very homogeneous range of BMI, and without any medication or overt chronic diseases except for metabolic syndrome (14), suggests that if ImP influences blood pressure, it may do so through mechanisms other than insulin resistance.

While the association between ImP and obesity, as well as blood pressure has been observed, the underlying mechanisms remain unclear. Future research should delve deeper into the molecular pathways linking ImP to obesity and hypertension and explore potential therapeutic targets to mitigate its effects on cardiovascular health. Additionally, the discrepancy in findings regarding the association between ImP and insulin resistance highlights the need for further clarification through well-controlled studies involving diverse populations.

## 4 Reduced ImP for cardiometabolic benefits? - insights from diet and gut health

Unhealthy dietary patterns, such as the contemporary Western diet characterized by low fiber content and high levels of animal proteins, saturated fats, sodium, and sugar, have been strongly linked to cardiometabolic disorders (65, 66). Conversely, diets rich in fiber and vegetable proteins, such as the Mediterranean, vegetarian, or plant-based low-protein diets, have shown metabolic benefits (67, 68). Increasing evidence suggests that metabolites produced by gut microbiota play a crucial role in mediating the effects of dietary patterns on host metabolism (69). In the MetaCardis study, circulating ImP levels were positively correlated with saturated fat intake (primarily driven by high cheese consumption) and negatively correlated with fiber and unsaturated fat intake (due to increased consumption of vegetables and nuts) (13). ImP levels were also inversely associated with dietary quality indices like the Alternate Healthy Eating Index, dietary diversity score, and Mediterranean diet scores (13). In a two-week dietary intervention study involving healthy individuals, transitioning from a Western diet to one rich in fiber, fruits, vegetables, and protein led to increased creatinine-normalized urinary ImP levels (70). Similarly, another intervention study focusing on subjects with HbA1c levels exceeding 6% showed that intake of resistant maltodextrin (a type of dietary fiber) reduced fecal ImP levels, particularly in individuals with elevated ImP levels before intervention (71). Overall, dietary fiber intake, unsaturated fat consumption, and adherence to healthy dietary patterns may inversely correlate with glucose metabolism disorders by reducing ImP levels.

Healthy dietary patterns, including high-fiber diets, are associated with greater intestinal microbial diversity and bacterial gene richness (72–74). Decreased intestinal microbial diversity and bacterial gene richness have been linked to metabolic disturbances (75–77). ImP has emerged as a circulating metabolite reflective of gut microbiome α diversity metrics, with a Shannon diversity index of approximately -0.2 (*P* < 0.01) (15), and is elevated in individuals with low bacterial gene richness (13). Thus, ImP may serve as an important biomarker reflecting the combined influence of diet, gut microbiota, and genetic diversity on cardiometabolic health.

As a natural source of SCFAs, slight differences in dietary fiber structure can lead to distinct effects on gut microbiome composition, resulting in targeted shifts in the production of SCFAs (78). Increased consumption of vegetables and fruits has been associated with higher abundance of SCFA-producing bacteria. For instance, fruit and vegetable intakes were positively associated with *Coprococcus* species, *Faecalibacterium prausnitzii*, R*oseburia hominis*, and *Firmicutes bacterium CAG:95* across multiple studies (79–83), which have been reported to have a significant negative correlation with ImP production, as depicted in **Table 1**. Additionally, studies indicate that decreased SCFA production leads to an increase in intestinal pH (16, 56), providing an optimal environment for urocanate reductase, the bacteria responsible for ImP production, to exert maximal activity. While current evidence is limited, given the observed link between decreased SCFAs and metabolic impairments in numerous studies, this hypothesis seems plausible, suggesting that ImP could serve as a biomarker of dysregulated gut microbiome—due to an unhealthy diet or disease. Therefore, implementing dietary modifications to promote healthier eating habits, along with interventions targeting the regulation of intestinal microbiota composition—such as fecal microbiota transplantation, specific microbiota transplantation or supplementation with probiotics/prebiotics—may offer effective strategies to reduce ImP levels. However, significant gaps remain in understanding the mechanistic relationship between ImP, SCFA production, gut microbiota composition, and intestinal environment. Future research endeavors should aim to elucidate these mechanisms and explore clinical interventions targeting dietary adjustments and microbiota modulation to mitigate ImP-related cardiometabolic risks.

## 5 Knowledge gaps and future perspectives

### 5.1 Development of novel drug targets associated with ImP

The association between elevated ImP levels and cardiovascular metabolic impairment underscores the importance of exploring the mechanisms underlying ImP’s effects, which may elucidate the potential for targeting ImP to improve cardiovascular metabolism and identify novel drug targets. Insights from the work of Koh and Molinaro et al. (12, 13, 36) have shed light on this field. Their research team elucidated the molecular mechanism by which ImP impairs glucose tolerance and insulin signaling through the activation of the p38γ/p62/mTORC1 signaling pathway. Further studies have revealed that ImP inhibits the hypoglycemic activity of metformin via the p38γ/Akt/AMPK pathway. Structural analysis has unveiled the interaction between ImP and the adenosine triphosphate (ATP) binding pocket of p38γ. In silico analysis suggests that pirfenidone, used to treat idiopathic pulmonary fibrosis, may compete with ImP for binding to this site of p38γ, implying its potential as a candidate for combination therapy in individuals with T2D who are unresponsive to metformin. Future research should focus on elucidating the detailed mechanism of ImP binding to p38γ for structure-based drug design. While pirfenidone shows promise, clinical trials are needed to confirm its efficacy and safety in T2D patients, along with investigations into potential drug interactions.

Besides opposing the interaction involving ImP, strategies aimed at reducing ImP production, like inhibiting enzymes such as urocanate reductase, present promising new therapeutic avenues for T2D. In this regard, Venskutonytė and Koh et al. elucidated the X-ray structures of its ligand-binding domains (26), providing valuable insights for structure-based drug design and aiding in the development of inhibitors for potential treatment of metabolic disorders.

### 5.2 Limitations and future directions

While the existing research provides valuable insights into the role of ImP in cardiometabolic diseases, several limitations and areas for future investigation should be acknowledged. Firstly, much of the current research focuses on observational and cross-sectional studies, limiting our ability to establish causal relationships between ImP and cardiometabolic diseases. Longitudinal follow-up studies are needed to elucidate the temporal relationship between ImP levels and disease onset and progression.

Secondly, there is a noticeable lack of diversity in study populations, with a predominant inclusion of individuals of European descent in many studies. Given that the gut microbiome exhibits significant variation across geographical regions (84, 85), and considering that the production of ImP is highly dependent on bacterial activity, it becomes imperative for future studies to encompass more diverse populations. This approach will not only enhance the generalizability of findings but also allow for a comprehensive understanding of how ethnic and geographic factors may influence ImP production and its implications for cardiometabolic diseases.

Thirdly, while circulating ImP levels have been correlated with systemic inflammation in metabolic disorder (13, 18, 20), the underlying mechanisms remain unclear, highlighting the necessity for further research into ImP’s regulation of inflammatory pathways. Notably, rectal administration of ImP in mice resulted in a significant increase in NF-κB, iNOS, and IL-6 expression, accompanied by a reduction in goblet cell count (23). These findings suggest ImP’s potential to induce intestinal inflammation, disrupt the intestinal barrier, and alter goblet cell proliferation. Given the crucial role of intestinal barrier integrity in cardiometabolic diseases, targeting ImP to modulate intestinal barrier function holds promise for cardiometabolic disease treatment. However, additional evidence is required to substantiate ImP’s regulatory role and elucidate its molecular mechanisms in this context.

Fourthly, optimal enzymatic activity of UrdA occurs under neutral pH conditions (28), while the proximal colon tends to maintain an acidic environment. Reduced production of SCFAs and increased protein fermentation in the gut contribute to the elevation of colonic pH (86), potentially facilitating ImP production. This inference partially elucidates the observed association between decreased fiber intake and increased ImP levels in certain populations, along with the correlation between a Western diet and elevated ImP content (13, 70, 71). Therefore, future research should focus on elucidating the contributions and mechanisms of factors influencing intestinal pH on ImP levels, thereby providing theoretical insights into targeting ImP-mediated pathways of cardiovascular metabolic damage. Additionally, the inhibitory effect of ImP on the hypoglycemic efficacy of metformin (36) underscores the profound impact of gut microbiota and their metabolites on drug therapy. Exploring the effects of drugs related to the treatment of cardiovascular metabolic diseases on ImP-producing bacteria and ImP content presents a novel and intriguing topic, offering potential new insights into the therapeutic mechanisms of drugs.

Furthermore, while diet has been shown to significantly influence ImP production via its effects on gut microbiota composition, it should be noted that other potential factors influencing ImP levels, such as genetic predisposition or medication use, have not yet been thoroughly investigated. Future research will be needed to explore these dimensions, as well as to further elucidate the mechanistic links between ImP and cardiometabolic diseases.

Lastly, as an increasing number of studies shed light on the correlation between gut bacteria and ImP production, as demonstrated in **Table 1**, it becomes evident that, like many other investigations focused on the gut microbiome, these studies raise more questions than they answer. Unveiling the precise contributions and mechanisms of these bacteria to ImP production is a vast and intricate endeavor, yet it constitutes a crucial aspect of research on ImP and warrants significant attention in future studies.

## 6 Conclusion

**Figure 1:**
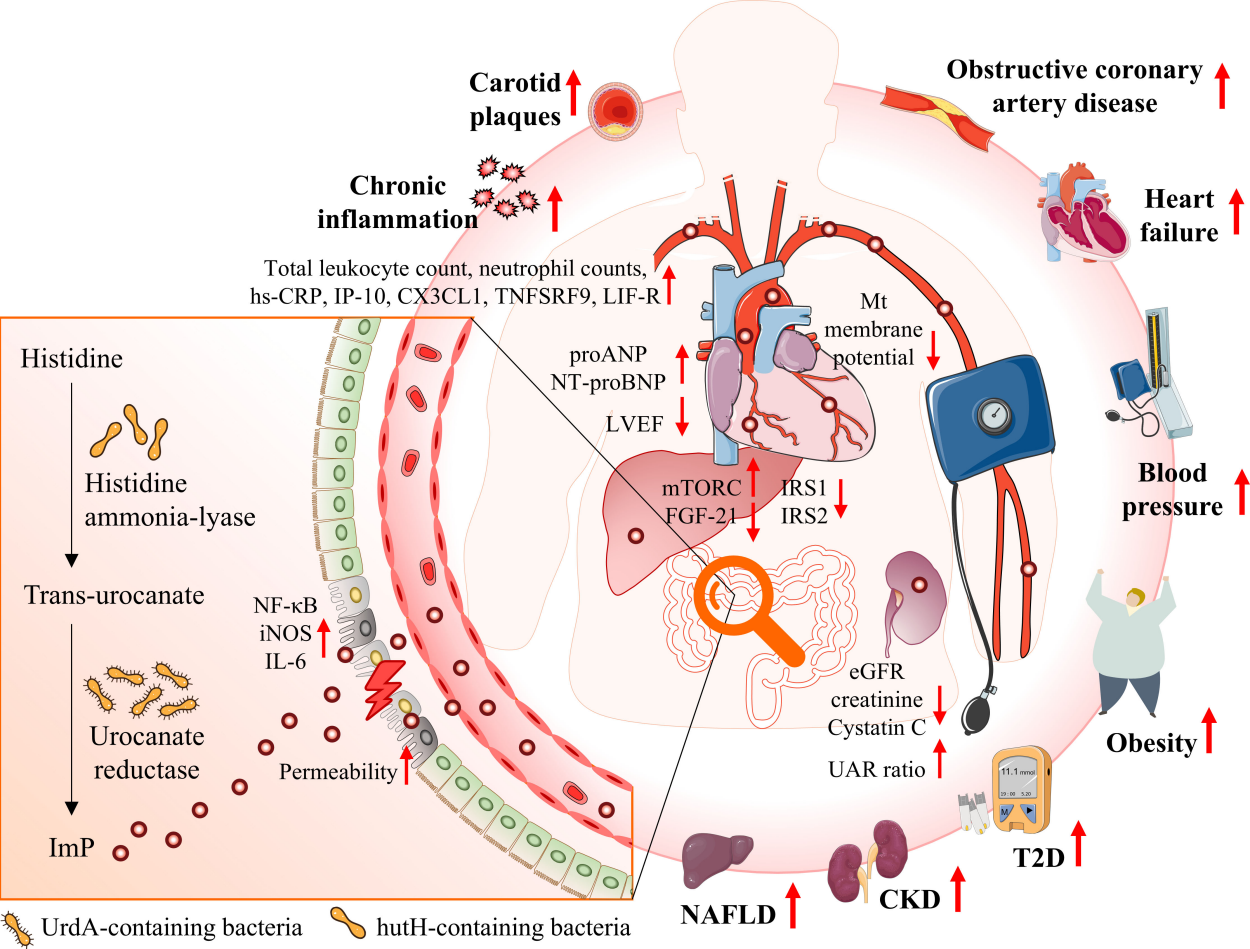
Illustrates the interplay between gut microbiota, ImP production, and cardiometabolic diseases. Epidemiological studies have identified a strong association between elevated ImP levels and an increased risk of onset of T2D and obesity, exacerbation of kidney traits in CKD, progression of atherosclerotic plaques, and elevated mortality rates in HF. ImP is a microbial metabolite derived from histidine via urocanate reductase, an enzyme encoded by the UrdA gene. This pathway is predominantly associated with certain gut bacteria. ImP may contribute to the heightened risk of cardiometabolic diseases through mechanisms such as impaired intestinal barrier function, activation of the p38γ/p62/mTORC1 signaling pathway, promoting systemic inflammation, and impairing cardiac and renal function. CKD, chronic kidney disease; eGFR, estimated glomerular filtration rate; HF, heart failure; ImP, imidazole propionate; IRS1, insulin receptor substrates 1; IRS2, insulin receptor substrates 2; MT, mitochondria; mTORC1, mechanistic target of rapamycin complex 1; NAFLD, non-alcoholic fatty liver disease; SCFAs, short-chain fatty acids; T2D, type 2 diabetes; UAC, urinary albumin-to-creatinine.

The interplay among cardiometabolic diseases underscores the importance of exploring shared metabolic pathways to enhance risk prediction and develop tailored prevention and management strategies. Over the past few years, multiple clinical studies have identified the intestinal metabolite ImP as a potential microbial signature linking insulin resistance, T2D, hypertension, obesity, NAFLD, CKD, atherosclerosis, and HF (**Table 2**, **Figure 1**). Our study emphasizes the significance of ImP as a potential biomarker and therapeutic target, highlighting the need for longitudinal research and diverse population participation to validate these associations. Future investigations should prioritize elucidating the molecular mechanisms underlying ImP’s role and its contribution to the pathogenesis of cardiometabolic diseases and related comorbidities, thereby advancing treatments for these conditions.

**Table 2:** The relationship between ImP and cardiometabolic disease/risk factors.

Disease	Clinical investigation		Sample	Main results	Reference
	Country	Research object			
T2D	Netherlands	15 obese subjects (BMI > 40): 5 with, 10 without T2D	Portal and peripheral plasma	ImP levels were higher in T2D subjects than in non-diabetic subjects (portal vein: *P* < 0.001; peripheral: *P* < 0.05).	(12)
	Sweden	649 subjects, aged 50-64: 335 NGT, 119 IFG, 142 IGT, and 53 naive T2D	Peripheral plasma	Treatment-naive T2D subjects had higher ImP levels than those with normal glucose tolerance, even after adjusting for BMI, sex, and age (men: 7.6 vs. 15.1 nM; women: 11.2 vs. 24.9 nM; adjusted P < 0.0001).	
T2D	Sweden	69 subjects aged over 65 with T2D, exclusively treated with metformin	Plasma	Metformin-treated subjects with blood glucose ≥7.8 mM had higher mean ImP levels than those with <7.8 mM (*P* < 0.05).	(36)
T2D	Europe (France, Germany, and Denmark)	1958 participants: 765 T2D, 654 prediabetes, and 539 healthy controls	Serum	1. ImP levels are higher in prediabetes and T2D compared to healthy controls (*P* < 0.001). 2. High ImP quartile (Q4) is associated with increased risks of prediabetes (OR = 1.75, *P* = 0.006) and T2D (OR = 2.76, P < 0.001). 3. ImP positively correlates with HbA1c, glycemia, insulinemia, HOMA-IR, and triglyceride-glucose index, and negatively with HOMA-B (*P* < 0.01, all). 4. Higher ImP levels are linked to increased glucose (*P* = 0.053), insulin (*P* = 0.02), and C-peptide after OGTT (*P* = 0.00014), with reduced Stumvoll sensitivity index (*P* = 0.004). 5. T2D subjects have higher ImP levels (28.1 nM) than those with prediabetes (27.8 nM) or normal glucose tolerance (19.7 nM) (*P* = 0.028). 6. CVD subjects have higher ImP levels (36.7 nM vs. 25.2 nM, *P* < 0.001). 7. Serum ImP positively correlates with inflammation markers (total leucocyte count, hs-CRP, IP-10) and negatively with circulating MAIT levels (*P* < 0.01, all).	(13)
T2D and Obesity	UK	1018 females	Serum	ImP serum levels exhibit positive correlations with BMI (r = 0.18, *P* = 5E-9), fasting glucose (r = 0.065, *P* = 0.05), and visceral fat mass (r = 0.067, *P* = 0.06), and are linked to increased risk of obesity (r = 0.2, P = 8E-9) and T2D (r = 0.095, *P* = 0.02).	(15)
Overweight and Obesity	Netherlands	107 participants (BMI > 25, medication-free for the last 3 months): 76 insulin-resistant, 31 insulin-sensitive	Plasma	1. ImP levels significantly correlated with diastolic blood pressure (r_s_ = 0.285, *P* = 0.004) and showed borderline significance with systolic blood pressure (r_s_ = 0.187, adjusted *P* = 0.060) and LDL-cholesterol (r_s_ = −0.181, adjusted *P* = 0.064); 2. Among overweight/obese non-T2D subjects, plasma ImP concentrations do not significantly differ between insulin-sensitive and insulin-resistant individuals (16.3 nM vs. 19 nM).	(14)
T2D	China	96 T2D subjects	Plasma	ImP levels significantly correlated with postprandial blood glucose (r = 0.249, *P* = 0.028)	(31)
T2D, CHF, and CKD	China	260 participants: 23 NGT, 48 NGT + CHF, 83 prediabetes + CHF, 56 prediabetes + CHF + CKD, 34 T2D + CHF, and 16 T2D + CHF + CKD	Serum	1. ImP is a common metabolite in patients with prediabetes/T2D, CHF, and CKD. 2. ImP levels increase 1.1–1.6 times with each additional CHF comorbidity (e.g., NGT vs. NGT + CHF: FC = 1.1, *P* = 0.047; T2D + CHF vs. NGT + CHF: FC = 1.6, *P* = 0.055; T2D + CHF + CKD vs. T2D + CHF: FC = 1.5, *P* = 0.011). 3. ImP strongly associates with CKD biomarkers, including creatinine, Cystatin C, and eGFR.	(21)
CVD and HF	Europe (France, Germany, and Denmark)	1985 participants: 133 HF, 282 CVD, and 1569 without CVD/HF (metabolic disease and healthy subjects)	Serum	1. HF patients had higher ImP levels than those with CVD (*P* < 0.01) or non-CVD/HF (P < 0.001). 2. The highest ImP quartile increased HF risk (adjusted OR = 3.02, *P* < 0.05). 3. Elevated ImP levels were associated with reduced LVEF (adjusted *P* < 0.001). 4. ProANP and NT-proBNP levels rose with higher ImP quartiles (*P* < 0.001).	(51)
	USA	2155 participants: 407 HF, 1331 CVD, and 417 without CVD/HF		1. Circulating ImP levels are associated with T2D (*P* < 0.01); 2. CVD and HF patients have higher ImP levels than those without (*P* < 0.001); 3. The highest ImP quartile significantly increases HF risk (adjusted OR = 2.89, *P* < 0.001); 4. Elevated ImP levels are associated with reduced LVEF (adjusted *P* < 0.01); 5. NT-proBNP levels rise with higher ImP quartiles (adjusted *P* < 0.0001); 6. High ImP (Q4) predicts higher mortality risk (adjusted HR = 1.85, *P* < 0.01); 7. ImP outperforms traditional predictors for 5-year mortality (*P* < 0.01).	
CKD	USA	2438 participants: 292 CKD (Stages 1–5: 134, 76, 73, 7, and 2, respectively), 2146 without CKD.	Serum	1. Longitudinal ImP elevation is linked to reduced eGFR (Beta = −1.33, 95% CI [−2.31, −0.35], *P* = 0.008); 2. Elevated ImP levels predict worsening kidney traits (lower eGFR, higher UAC ratio, CKD) over ~6 years, especially in diabetics.	(17)
Atherosclerosis	USA	493 females with or at high risk of HIV infection: 84 with plaque, 236 without plaque	Plasma	1. Plasma ImP positively associates with carotid artery plaque (*P* = 0.043). 2. ImP positively correlates with inflammatory markers (CX3CL1, TNFSRF9, LIF-R; *P* < 0.05) 3. ImP-associated gut bacterial score correlates significantly with higher plasma ImP levels and increased plaque odds (OR = 1.31).	(18)
Atherosclerosis	Denmark	254 HIV-infected participants: 60 obstructive CAD, 80 nonobstructive CAD, 114 without CAD	Plasma	1. Plasma ImP levels were higher in obstructive CAD patients compared to nonobstructive CAD (*P* = 0.022) and no CAD (*P* = 0.00047). 2. High ImP levels (Q4) increased obstructive CAD odds in univariable analysis (OR = 2.3, *P* = 0.01) but not after adjustment (*P* > 0.05).	(19)
Cardiometabolic health	USA	446 females: 300 HIV+, 146 HIV−	Plasma	ImP levels positively correlate with HbA1C (*P* < 0.01) and negatively with FGF-21 (rs = -0.229, *P* < 0.001).	(30)

BMI, body mass index; CAD, coronary artery disease; CHF, chronic heart failure; CKD, chronic kidney disease; CVD, cardiovascular disease; eGFR, estimated glomerular filtration rate; FC, fold change; HbA1C, glycated hemoglobin; HF, heart failure; HIV, human immunodeficiency virus; HOMA-IR, homeostatic model assessment of insulin resistance; HR, hazard ratio; hs-CRP, C-reactive protein; IFG, impaired fasting glucose; IGT, impaired glucose tolerance; ImP, imidazole propionate; IP-10, interferon gamma-induced protein 10; LDL, low-density lipoprotein; LVEF, left ventricular ejection fraction; MAIT, mucosal-associated invariant T cells; NGT, normal glucose tolerance; NT-proBNP, N-terminal pro–B-type natriuretic peptide; OGTT, oral glucose tolerance tests; OR, odds ratio; proANP, pro-atrial natriuretic peptide; r, Pearson correlation coefficient; rs, Spearman rank correlation coefficient; T2D, type 2 diabetes; UAC, urinary albumin-to-creatinine.
